# Hepatitis E Virus Infections in Free-Ranging and Captive Cetaceans, Spain, 2011–2022

**DOI:** 10.3201/eid2812.221188

**Published:** 2022-12

**Authors:** Javier Caballero-Gómez, Antonio Rivero-Juarez, Adrián Beato-Benítez, Carolina Fernández-Maldonado, Mariano Domingo, Daniel García-Párraga, Antonio Fernández, Eva Sierra, Rainer G. Ulrich, Eva Martínez-Nevado, Cecilia Sierra-Arqueros, Rocío Canales-Merino, Antonio Rivero, Ignacio García-Bocanegra

**Affiliations:** Maimonides Institute for Biomedical Research of Cordoba, Reina Sofía University Hospital, University of Córdoba, Córdoba, Spain (J. Caballero-Gómez, A. Rivero-Juarez, A. Rivero); (J. Caballero-Gómez, A. Rivero-Juarez, A. Rivero, I. García-Bocanegra);; GISAZ-ENZOEM, University of Córdoba, Córdoba (J. Caballero-Gómez, A. Beato-Benítez, I. García-Bocanegra);; CIBERINFEC, Carlos III Health Institute, Madrid, Spain Seashore Environment and Fauna, Cádiz, Spain (C. Fernández-Maldonado);; Andalusian Marine Environment Management Center, Ministry of Agriculture, Livestock, Fisheries and Sustainable Development, Cádiz, Spain (C. Fernández-Maldonado);; Veterinary Pathology Diagnostic Service, Autonomous University of Barcelona-Bellaterra, Barcelona, Spain (M. Domingo);; Oceanographic Foundation of the Valencian Community and Avanqua Oceanographic, Valencia, Spain (D. García-Párraga);; Atlantic Cetacean Research Center, Institute of Animal Health, University of Las Palmas de Gran Canaria, Trasmontaña, Las Palmas, Spain (A. Fernández, E. Sierra);; Federal Research Institute for Animal Health/German Center for Infection Research, Greifswald-Insel Riems, Germany (R.G. Ulrich);; Madrid Zoo, Madrid, Spain (E. Martínez-Nevado);; Selwo Marina, Málaga, Spain (C. Sierra-Arqueros);; Mundomar Benidorm, Alicante, Spain (R. Canales-Merino).

**Keywords:** Hepatitis E, cetaceans, Spain, viruses, zoonoses, hepatitis E virus

## Abstract

Epidemiologic surveillance of hepatitis E virus in over 300 free-ranging and captive cetaceans in waters off Spain revealed extensive exposure to this pathogen. We suggest the persistent and widespread presence of hepatitis E in the marine environment off the coast of Spain may be driven by terrestrial sources of contamination.

*Paslahepevirus balayani* (previously known as hepatitis E virus [HEV]; family *Hepeviridae*) is the leading cause of acute viral hepatitis in humans (*1*,*2*). Although 8 different genotypes of HEV have been identified, HEV-3 is the genotype with the broadest geographic distribution, including Europe, where the number of hepatitis E cases has sharply increased in the past decade (*3*). The main reservoirs of this genotype are suids, but a wide range of other land mammals has been shown to be susceptible to this emerging genotype (*2*). Although echinoderms and several bivalve shellfish species from coastal waters have tested positive for HEV, the susceptibility of other marine animals, such as cetaceans, to HEV has been unknown, as has their possible role in the epidemiology of this family of viruses (*4*). We conducted a large-scale study to determine the seroprevalence and prevalence of HEV in cetacean populations, both free-ranging and captive, in Spain, and to assess the dynamics of seropositivity in marine animals sampled longitudinally during the study period.

## The Study

**Figure 1:**
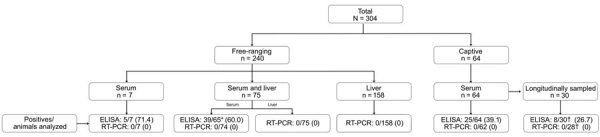
Flowchart of a survey of hepatitis E virus in 304 cetaceans belonging to 13 species in Spain during 2011–2022. Description of the study population, number of cetaceans, type of samples analyzed by ELISA and RT-PCR, and results obtained in each assay. *Ten of 75 serum samples were discarded for serologic analysis due to hemolysis. †Taking into account that 2–5 samples were collected per animal in longitudinally surveyed animals, 97 were analyzed by ELISA and 78 by RT-PCR. RT-PCR, reverse transcription PCR.

We collected blood and liver samples from 304 cetaceans belonging to 13 different species in Spain during 2011–2022 (Table 1; Figure 1). We based our study on 240 free-ranging animals found stranded on the Atlantic and Mediterranean coasts of Spain and 64 cetaceans kept in captivity at 6 aquatic parks (deemed A–F) in Spain. We performed longitudinal surveillance on 30 of the 64 animals kept in aquatic parks during the study period.

**Table 1:** Distribution of hepatitis E virus seroprevalence in free-ranging and captive cetacean populations in Spain and results of bivariate analysis*

Variable	Categories	Free-ranging			Captive	
		No. positive/no. analyzed (% positive)†	p value		No. positive/no. analyzed (% positive)†	p value
Species‡	Atlantic spotted dolphin (*Stenella frontalis*)	1/1 (100.0)	0.191		NA	0.323
	Beluga (*Delphinapterus leucas*)	NA			0/2 (0.0)	
	Bottlenose dolphin (*Tursiops truncatus*)	0/2 (0.0)			21/55 (38.2)	
	Common dolphin (*Delphinus delphis*)	1/2 (50.0)			NA	
	Cuvier’s beaked whale (*Ziphius cavirostris*)	1/1 (100.0)			NA	
	Killer whale (*Orcinus orca*)	NA			4/7 (57.1)	
	Risso’s dolphin (*Grampus griseus*)	3/8 (37.5)			NA	
	Southern long-finned pilot whale (*Globicephala melas*)	0/1 (0.0)			NA	
	Striped dolphin (*Stenella coeruleoalba*)	38/57 (66.7)			NA	
Age§	Adult	33/45 (73.3)	0.006		20/47 (42.6)	0.406
	Young	11/27 (40.7)			4/12 (33.3)	
Sex	F	25/39 (64.1)	0.373		12/33 (36.4)	0.485
	M	19/33 (57.6)			12/30 (40.0)	

*Analyses by pearson’s χ^2^ or Fisher exact test. NA, not applicable. †Animals with missing information excluded. ‡Samples from harbor porpoises (*Phocoena phocoena*), fin whales (*Balaenoptera physalus*), minke whales (*Balaenoptera acutorostrata*). and humpback whales (*Megaptera novaeangliae*) were also included in the study but were only tested by PCR. §Age was classified using the mean reproductive age of each species.

We assessed the presence of HEV antibodies in serum or plasma using a commercial multispecies ELISA (MP Biomedicals, https://www.mpbio.com) and, whenever possible, further investigated seropositivity by Western blot analysis (Appendix). We determined the presence of HEV RNA by using 2 broad-spectrum reverse transcription PCR (RT-PCR) assays in parallel (Appendix) (*5*,*6*). We analyzed associations between the presence of HEV antibodies and explanatory variables using Pearson χ^2^ test or Fisher exact test and further included variables with p<0.05 in the bivariate analysis (except habitat status) in a generalized estimating equation model.

**Figure 2:**
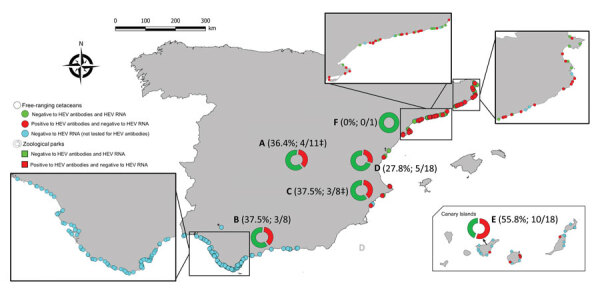
Spatial distribution of cetaceans sampled in a survey of HEV in 304 cetaceans belonging to 13 species in Spain during n 2011–2022. The frequency of seropositivity and number of seropositive and total animals analyzed at each zoological park (A–F) is shown in parentheses. Callouts show detail of sampling along the Atlantic and Mediterranean coastlines. *Animal sampled in the Guadalquivir River. †This animal was not analyzed by reverse transcription PCR. ‡One of the sampled animals of this zoo park was not tested by reverse transcription PCR. HEV, hepatitis E virus.

We identified 69 (50.7%, 95% CI 42.3%–59.1%) of 136 cetaceans as harboring anti-HEV antibodies (Table 1; Figures 1, 2; Appendix Table 1). We confirmed antibodies against HEV-3 in 5 of the 7 ELISA-positive animals analyzed by Western blot analysis: a free-ranging striped dolphin, a free-ranging Cuvier’s beaked whale, a free-ranging Risso’s dolphin, and 2 captive bottlenose dolphins. We found none (0.0%; 95% CI 0.0%–1.2%) of the 302 animals analyzed to be positive for HEV RNA (Figure 2).

We noted seroprevalence to be significantly higher in free-ranging animals (44/72; 61.1%; 95% CI 49.9%–72.4%) than in those kept in captivity (25/64; 39.1%; 95% CI 27.1%–51.0%) (relative risk = 2.5, 95% CI 1.2%–4.9%; p = 0.008). We found seropositivity in adult free-ranging cetaceans (33/45; 73.3%) to be significantly higher than that in young animals (11/27; 40.7%; odds ratio 4.0, 95% CI 1.4–11.0; p = 0.006).

Our testing revealed seropositive animals in 5 of the 6 aquatic facilities sampled; within-zoo seroprevalence ranged from 27.8% in aquatic park D to 55.6% in aquatic park E (Table 2; Figure 2). Of the 30 longitudinally sampled animals, 21 remained seronegative, and 6 animals showed seropositivity at all samplings during the study period (Appendix Table 2). Two bottlenose dolphins seroconverted, 1 in 2013 and another in 2017. Seroreversions were detected in 2 animals (1 a dolphin that had shown seroconversion); 1 incident occurred 1 year after the first positive sampling, the other 5 years.

**Table 2:** Distribution of hepatitis E virus seroprevalence in cetaceans in Spain, by sampling location, and results of bivariate analyses

Category	No. positive/no. analyzed (% positive)	p value
Free-ranging areas		
Atlantic Ocean	6/8 (75.0)	0.327
Mediterranean Sea	38/64 (59.4)	
Aquatic parks		
A	4/11 (36.4)	0.772
B	3/8 (37.5)	
C	3/8 (37.5)	
D	5/18 (27.8)	
E	10/18 (55.6)	
F	0/1 (0.0)	

## Conclusions

Our survey reveals high exposure to HEV in free-ranging and captive populations of cetaceans in Spain. The detection of HEV antibodies in Atlantic spotted, common, Risso’s, and striped dolphins, as well as in Cuvier’s beaked and killer whales, demonstrates an increase in the number of cetartiodactyls susceptible to this virus (*2*).

Ingestion of contaminated food is considered to be one of the main transmission routes of HEV in humans and has also been suggested for other mammal species, including dolphins (*4*). The seropositive species detected in our study feed on a wide variety of resources, including fish and cephalopods. The presence of HEV in these food resources has not yet been assessed, but the virus has been frequently detected in such other aquatic animals as sea urchins and bivalve shellfish in different areas of Europe (*2*,*7*), which provides evidence that HEV does abide in marine ecosystems. Of note, the virus is shed primarily in the feces of infected species, which can lead to viral contamination of the environment, and HEV has been shown to be highly resistant to even high concentrations of salt (*8*). Contaminated water has been considered a potential source of zoonotic HEV (*9*), because drinking tap water or water from private wells or nearby rivers has been suggested as a risk factor for acquiring HEV infection in humans (*10*). This hypothesis is supported by a study conducted in captive cetaceans all sharing the same tanks, which revealed the detection of seropositivity and active HEV infection (*4*).

The significantly higher seroprevalence we found in adult free-ranging animals compared with young animals likely reflects the increased cumulative exposure to HEV in these species. Our additional discovery of HEV antibodies in 4 free-ranging yearlings in 2011, 2019, and 2021 could suggest endemic circulation of HEV in cetaceans living in Spanish waters during the study period. Free-ranging cetaceans had a 2.5-times higher risk of being exposed to HEV than those kept in captivity, which might be explained by differences in diet or longer exposure to environmental contamination. Human- and swine-related HEV-3 strains have been detected in sewage and slurry in Spain (*11*) and in rivers and coastal waters in Italy (*12*). The high census of some susceptible domestic and wildlife species (*13*,*14*), combined with high coastal urbanization and insufficient control of urban sewage in some regions of our study area (*15*), might be contributing factors in the higher seropositivity we noted in free-ranging cetaceans. By contrast, cetaceans in zoological parks, including those analyzed in our study, live in large water tanks that are frequently decontaminated with ozone, ultraviolet radiation, brine, or chlorine, some of which deactivates HEV (*9*). Nonetheless, the high seroprevalence we observed in the 5 zoos with seropositive animals indicates a wide circulation of the virus in these more controlled environments.

The 2 seroconversions we noted in captive bottlenose dolphins support the hypothesis of HEV circulation in zoos during our study period. However, 4 of the longitudinally surveyed cetaceans remained seropositive at all samplings. This finding might be due to the long-lived persistence of anti-HEV antibodies in cetaceans, which is supported by the significantly higher seroprevalence we detected in older, free-ranging cetaceans. There is no known information about the long-term persistence of HEV antibodies in these species. Thus, possible loss of antibodies and reexposure in some of the persistently seropositive cetaceans during the study period cannot be ruled out, as evidenced by the seroreversions we observed in 2 bottlenose dolphins 1 and 5 years after the first seropositive sampling was detected.

In conclusion, the seropositivity noted in our study indicates widespread circulation of HEV in both free-ranging and captive cetacean populations in southwestern Europe. Additional molecular and serologic studies are warranted to determine the role of cetaceans in the epidemiology of HEV and to elucidate the sources of HEV infection, particularly in the free-ranging cetacean population.

Appendix More information on hepatitis E virus infections in free-ranging and captive cetaceans, Spain, 2011–2022.
