# Supplementary material for: Hepatitis E Virus Infections in Free-Ranging and Captive Cetaceans, Spain, 2011–2022
Source: Emerg Infect Dis. 2022 Dec;28(12):2543–7. doi: 10.3201/eid2812.221188 (PMC9707570; doi:10.3201/eid2812.221188)
Supplement: Appendix — More information on hepatitis E virus infections in free-ranging and captive cetaceans, Spain, 2011–2022. [file 22-1188-Techapp-s1.pdf]

Article DOI: https://doi.org/10.3201/eid2812.221188

Hepatitis E Virus Circulation in Free-Ranging and Captive Cetaceans, Spain, 2011–2022

Appendix

Sampling

Serum or plasma samples were obtained by blood centrifugation at 400x *g* for 15 min. The median (Q1-Q3) interval between consecutive samplings of the follow-up was 35 months (range 22-118.5). Samples were stored at −20°C until laboratory analyses. Epidemiological data, including species, age, gender, habitat status (free-ranging *vs* under human care), sampling location (free-range areas [Atlantic Ocean *vs* Mediterranean Sea] and zoological institutions), sampling date and georeferenced location, were gathered from each animal. Whenever possible, both serological and molecular assays were conducted in serum samples (Table 1).

Serological analyses

The presence of anti-HEV antibodies was assessed using a commercial ELISA (HEV 4.0v; MP Diagnostics, Illkirch, France), which is based on the highly conserved and recombinant protein ET2.1 (*1*) and detects total antibodies against this virus in serum or plasma samples from a wide range of animal species. The cut-off was calculated using the formula: 0.2 + mean optical density (OD) of negative controls. In addition, sample results were expressed as an ELISA percentage (E%), calculated as follows: [E% = (sample OD)/(cut-off) × 100]. Longitudinally surveyed animals were considered seropositive if at least one serum sample tested positive by ELISA.

Whenever possible, samples from seropositive cetaceans were further investigated by western blot (WB) analysis to confirm exposure to *Paslahepevirus balayi* and/or *Rocahepevirus ratti* species, including HEV-3 and HEV-C1 genotypes, in cetaceans. In this connection,

Rocahepevirus ratti is other hepevirus whose zoonotic potential has recently been confirmed both by the detection of viral RNA in human hepatitis E cases and by experimental transmission to non-human primates (*2*,*3*). For WB analyses, carboxy-terminal segments of the capsid proteins of HEV-3 and HEV-C1, and a nucleocapsid protein derivative (amino acid residues 1-39/213-134 433) of the *Puumala orthohantavirus* strain Vranica/Hällnäs as negative control, were produced as His-tagged recombinant proteins in *Escherichia coli* and purified by nickel-chelate affinity chromatography (*4*,*5*). Seropositivity was confirmed by WB when blot bands matching either HEV-3 or HEV-C1 antigens or both were observed, but without reactivity to the negative control antigen. The presence of specific antibodies against HEV-3 or HEV-C1 was considered when samples reacted against the capsid protein derivative of only one of these genotypes, otherwise the result was considered indeterminate.

Molecular analyses

RNA was extracted from serum/plasma and liver samples using the QIAmp MinElute Virus Spin and RNeasy Mini kits (QIAGEN, Hilden, Germany), respectively. Liver RNA samples were extracted individually, whereas RNA from serum/plasma samples was obtained using pools of four samples (total volume: 400µl). A real-time RT-PCR (CFX Connect Real Time PCR System) that detects all *Paslahepevirus balayani* genotypes (HEV-1 to HEV-8) was performed using 25µl of RNA template and the QIAGEN One-Step RT-PCR kit, as previously described (*6*). The detection limit was set at 21.9 IU/mL (95% Confidence Interval (95% CI): 17.4-34.3). A nested broad-spectrum RT-PCR (Fisher Scientific Applied Biosystems SimpliAmp™) capable of detecting the four genera of hepevirus was carried out using the QIAGEN One-Step RT-PCR kit for the first round of RT-PCR, and a premixed 2X solution containing Taq DNA Polymerase, dNTPs and reaction buffer (Promega, Madison, WI, USA) for the second round (*7*). The nested RT-PCR amplicons were examined on 1.5% agarose gels stained with RedSafe™ Nucleic Acid Staining solution (iNtRON Biotechnology, Seongnam, Korea). The WHO HEV-3a reference strain (code 6329/10), supplied by the Paul-Ehrlich Institut, was included as positive control in each run of the two RT-PCR assays used.

Appendix Table 1. Anti-HEV antibody-positive samples in free-ranging cetacean populations and those under human care in Spain, 2011–2022. Samples from longitudinally surveyed animals are labeled with the same number and consecutive letters.

ID	Species	Habitat status	Sampling location	E%
1	Striped dolphin	Free-ranging	Mediterranean Sea	345,82
7	Striped dolphin	Free-ranging	Mediterranean Sea	305,18
8	Striped dolphin	Free-ranging	Mediterranean Sea	1786,45
9	Striped dolphin	Free-ranging	Mediterranean Sea	1699,60
10	Striped dolphin	Free-ranging	Mediterranean Sea	174,90
12	Striped dolphin	Free-ranging	Mediterranean Sea	1680,48
14	Risso's dolphin	Free-ranging	Mediterranean Sea	767,73
15	Striped dolphin	Free-ranging	Mediterranean Sea	958,96
16	Striped dolphin	Free-ranging	Mediterranean Sea	1821,51
18	Striped dolphin	Free-ranging	Mediterranean Sea	475,70
20	Striped dolphin	Free-ranging	Mediterranean Sea	733,86
22	Striped dolphin	Free-ranging	Mediterranean Sea	1803,19
25	Striped dolphin	Free-ranging	Mediterranean Sea	105,98
26	Striped dolphin	Free-ranging	Mediterranean Sea	1684,06
28	Striped dolphin	Free-ranging	Mediterranean Sea	381,67
29	Striped dolphin	Free-ranging	Mediterranean Sea	1647,81
30	Striped dolphin	Free-ranging	Mediterranean Sea	1882,47
33	Striped dolphin	Free-ranging	Mediterranean Sea	1715,94
35	Striped dolphin	Free-ranging	Mediterranean Sea	578,49
37	Striped dolphin	Free-ranging	Mediterranean Sea	325,50
38	Striped dolphin	Free-ranging	Mediterranean Sea	1595,62
40	Striped dolphin	Free-ranging	Mediterranean Sea	1279,68
45	Striped dolphin	Free-ranging	Mediterranean Sea	515,14
49	Striped dolphin	Free-ranging	Mediterranean Sea	288,45
50	Striped dolphin	Free-ranging	Mediterranean Sea	163,75
51	Striped dolphin	Free-ranging	Mediterranean Sea	1705,58
55	Striped dolphin	Free-ranging	Mediterranean Sea	1768,53
56	Risso's dolphin	Free-ranging	Mediterranean Sea	290,84
59	Striped dolphin	Free-ranging	Mediterranean Sea	1777,29
60	Striped dolphin	Free-ranging	Mediterranean Sea	707,57
64	Striped dolphin	Free-ranging	Mediterranean Sea	1191,63
65	Striped dolphin	Free-ranging	Mediterranean Sea	921,91
66	Striped dolphin	Free-ranging	Mediterranean Sea	1656,18
67	Striped dolphin	Free-ranging	Mediterranean Sea	778,37
69	Striped dolphin	Free-ranging	Mediterranean Sea	474,69
70	Striped dolphin	Free-ranging	Mediterranean Sea	293,06
74	Striped dolphin	Free-ranging	Mediterranean Sea	1731,43
75	Striped dolphin	Free-ranging	Mediterranean Sea	1595,92
78	Striped dolphin	Free-ranging	Atlantic Ocean	1861,84
79	Cuvier's beaked Whales	Free-ranging	Atlantic Ocean	1255,58
83	Striped dolphin	Free-ranging	Atlantic Ocean	706,07
84	Risso's dolphin	Free-ranging	Atlantic Ocean	500,59
87	Short-beaked common dolphin	Free-ranging	Atlantic Ocean	420,74
101	Atlantic spotted dolphin	Free-ranging	Atlantic Ocean	455,97
581	Bottlenose dolphin	Captivity	A	1700,60
583a	Bottlenose dolphin	Captivity	A	1136,33
583b	Bottlenose dolphin	Captivity	A	1187,35
583c	Bottlenose dolphin	Captivity	A	1102,45
587a	Bottlenose dolphin	Captivity	A	1633,06
594a	Bottlenose dolphin	Captivity	A	1598,79
594b	Bottlenose dolphin	Captivity	A	1470,20
1134	Bottlenose dolphin	Captivity	B	1006,12
1137	Bottlenose dolphin	Captivity	B	124,08
1138	Bottlenose dolphin	Captivity	B	1564,90
1187b	Bottlenose dolphin	Captivity	C	1616,33
1193a	Bottlenose dolphin	Captivity	C	220,50
1193b	Bottlenose dolphin	Captivity	C	178,72
1198	Bottlenose dolphin	Captivity	C	453,88
387a	Bottlenose dolphin	Captivity	D	117,99
392a	Bottlenose dolphin	Captivity	D	231,02
392b	Bottlenose dolphin	Captivity	D	264,49
392c	Bottlenose dolphin	Captivity	D	586,94
394a	Bottlenose dolphin	Captivity	D	225,31
394b	Bottlenose dolphin	Captivity	D	306,94
394c	Bottlenose dolphin	Captivity	D	177,14
399c	Bottlenose dolphin	Captivity	D	947,39
399d	Bottlenose dolphin	Captivity	D	389,64

ID	Species	Habitat status	Sampling location	E%
399e	Bottlenose dolphin	Captivity	D	351,84
1418b	Bottlenose dolphin	Captivity	D	233,47
1418c	Bottlenose dolphin	Captivity	D	264,60
81	Bottlenose dolphin	Captivity	E	763,64
1419	Bottlenose dolphin	Captivity	E	1144,24
1424	Bottlenose dolphin	Captivity	E	1608,48
1427	Bottlenose dolphin	Captivity	E	1397,17
1429	Bottlenose dolphin	Captivity	E	1690,91
82a	Killer whale	Captivity	E	125,64
82b	Killer whale	Captivity	E	227,47
1421	Killer whale	Captivity	E	217,37
1425	Killer whale	Captivity	E	187,47
1426	Killer whale	Captivity	E	480,00
1432	Killer whale	Captivity	E	150,30

E%, ELISA percentage; calculated as follows: [E% = (sample OD)/(cut-off) × 100]

Appendix Table 2. Antibodies against hepatitis E virus in longitudinally sampled cetaceans in Spain, 2011–2022. Dots indicate antibodies to hepatitis E virus (hollow: positive; solid: negative). When 2 samplings were carried out in the same year, abbreviated months are indicated.

ID	Species	Zoo	Interpretation	2009	2010	2013	2016	2017	2018	2019	2020	2021
383	Beluga	D	Seronegative at all samplings					●			●	
584	Bottlenose dolphin	A	Seronegative at all samplings					●		●		
586	Bottlenose dolphin	A	Seronegative at all samplings					●			●	
583	Bottlenose dolphin	A	Seropositive at all samplings					○		○	○	
594	Bottlenose dolphin	A	Seropositive at all samplings					○		○		
589	Bottlenose dolphin	A	Seronegative at all samplings				●Apr ●Jun					
595	Bottlenose dolphin	A	Seronegative at all samplings					●		●		
591	Bottlenose dolphin	A	Seronegative at all samplings					●		●		
592	Bottlenose dolphin	A/B	Seronegative at all samplings					●			●	
593	Bottlenose dolphin	A/B	Seronegative at all samplings					●			●	
1188	Bottlenose dolphin	C	Seronegative at all samplings							●	●	
1193	Bottlenose dolphin	C	Seropositive at all samplings							○	○	
1194	Bottlenose dolphin	C	Seronegative at all samplings							●		●
1199	Bottlenose dolphin	C	Seronegative at all samplings							●		●
399	Bottlenose dolphin	D	Seroconversion			●Aug ●Nov		○	○		○	
394	Bottlenose dolphin	D	Seropositive at all samplings					○	○		○	
392	Bottlenose dolphin	D	Seropositive at all samplings					○	○		○	
1418	Bottlenose dolphin	D	Seroconversion & Seroreversion	●		○		○	●		●	
387	Bottlenose dolphin	D	Seroreversion					○	●		●	
388	Bottlenose dolphin	D	Seronegative at all samplings		●	●		●	●		●	
389	Bottlenose dolphin	D	Seronegative at all samplings		●	●		●	●		●	
390	Bottlenose dolphin	D	Seronegative at all samplings		●	●		●	●		●	
391	Bottlenose dolphin	D	Seronegative at all samplings		●	●		●	●		●	

ID	Species	Zoo	Interpretation	2009	2010	2013	2016	2017	2018	2019	2020	2021
393	Bottlenose dolphin	D	Seronegative at all samplings		●	●		●	●		●	
395	Bottlenose dolphin	D	Seronegative at all samplings		●	●		●	●		●	
398	Bottlenose dolphin	D	Seronegative at all samplings					●	●			
396	Bottlenose dolphin	D	Seronegative at all samplings		●	●		●	●		●	
400	Bottlenose dolphin	D	Seronegative at all samplings	●		●		●	●		●	
397	Bottlenose dolphin	D	Seronegative at all samplings		●	●		●	●		●	
82	Bottlenose dolphin	E	Seropositive at all samplings									○Feb ○Jun

Apr, April; Jun, June; Aug, August; Nov, November; Feb, February

References

1. Hu WP, Lu Y, Precioso NA, Chen HY, Howard T, Anderson D, et al. Double-antigen enzyme-linked immunosorbent assay for detection of hepatitis E virus-specific antibodies in human or swine sera. Clin Vaccine Immunol. 2008;15:1151–7. PubMed https://doi.org/10.1128/CVI.00186-07

2. Sridhar S, Yip CCY, Wu S, Cai J, Zhang AJX, Leung KH, et al. Rat hepatitis E virus as cause of persistent hepatitis after liver transplant. Emerg Infect Dis. 2018;24:2241–50. PubMed https://doi.org/10.3201/eid2412.180937

3. Yang F, Li Y, Li Y, Jin W, Duan S, Xu H, et al. Experimental cross-species transmission of rat hepatitis E virus to rhesus and cynomolgus monkeys. Viruses. 2022;14:293. PubMed https://doi.org/10.3390/v14020293

4. Dremsek P, Wenzel JJ, Johne R, Ziller M, Hofmann J, Groschup MH, et al. Seroprevalence study in forestry workers from eastern Germany using novel genotype 3- and rat hepatitis E virus-specific immunoglobulin G ELISAs. Med Microbiol Immunol (Berl). 2012;201:189–200. PubMed https://doi.org/10.1007/s00430-011-0221-2

5. Lundkvist A, Meisel H, Koletzki D, Lankinen H, Cifire F, Geldmacher A, et al. Mapping of B-cell epitopes in the nucleocapsid protein of *Puumala hantavirus.* Viral Immunol. 2002;15:177–92. PubMed https://doi.org/10.1089/088282402317340323

6. Frías M, López-López P, Zafra I, Caballero-Gómez J, Machuca I, Camacho Á, et al. Development and clinical validation of a pangenotypic PCR-based assay for the detection and quantification of hepatitis E virus (*Orthohepevirus A* genus). J Clin Microbiol. 2021;59:e02075–20. PubMed https://doi.org/10.1128/JCM.02075-20

7. Johne R, Plenge-Bönig A, Hess M, Ulrich RG, Reetz J, Schielke A. Detection of a novel hepatitis E-like virus in faeces of wild rats using a nested broad-spectrum RT-PCR. J Gen Virol. 2010;91:750–8. PubMed https://doi.org/10.1099/vir.0.016584-0
